# Correlation between Nafion Morphology in Various Dispersion Liquids and Properties of the Cast Membranes

**DOI:** 10.3390/membranes13010013

**Published:** 2022-12-22

**Authors:** Ekaterina Yu. Safronova, Daria Yu. Voropaeva, Dmitry V. Safronov, Nastasia Stretton, Anna V. Parshina, Andrey B. Yaroslavtsev

**Affiliations:** 1 N. S. Kurnakov Institute of General and Inorganic Chemistry, Russian Academy of Sciences, 119991 Moscow, Russia; 2 Department of Analytical Chemistry, Voronezh State University, 394018 Voronezh, Russia

**Keywords:** nafion membrane, polymer dispersion, film casting, proton conductivity, gas permeability, selectivity

## Abstract

Nafion is a perfluorosulfonic acid polymer that is most commonly used in proton-exchange membrane fuel cells. The processes of pretreatment and formation of such membranes strongly affect their properties. In this work, dispersions of Nafion in various ionic forms and dispersing liquids (ethylene glycol, *N,N*-dimethylformamide, *N*-methyl-2-pyrrolidone and isopropyl alcohol–water mixtures in different ratios) were obtained and studied. Membranes fabricated by casting of the various dispersions were also studied. The effect of the nature of the dispersing liquid and the counterion on the properties of Nafion dispersions, the morphology of the polymer in the dispersions and the characteristics of the membranes obtained from them has been shown. Based on the overall results, it can be concluded that the use of perfluorosulfonic acid dispersions in aprotic polar solvents is advisable for obtaining membranes by the casting procedure. This is because it provides optimal polymer morphology in the dispersion, which leads to the formation of films with good selectivity, mechanical and transport properties. The performed investigations show the relationship between the composition of dispersions, the morphology of the polymer and the properties of the membranes formed from them by the casting procedure.

## 1. Introduction

Perfluorosulfonic acid polymer Nafion^®^ (Chemours^TM^) was obtained more than 50 years ago to prevent reversed product diffusion in chlorine and alkali production during the electrolysis of sodium chloride solutions [1]. Perfluorosulfonic acid membranes have high ionic conductivity, and high chemical, thermal and mechanical stability of the polymer in comparison to linear and aromatic hydrocarbon polymers [2]. Due to the set of such unique properties, Nafion^®^ membranes have been used as an electrolyte in low-temperature proton-exchange membrane fuel cells (PEMFC) since the 1990s [3,4,5,6].

Initially, perfluorosulfonic acid membranes were produced by extrusion techniques with thicknesses of >150 μm [7]. However, requirements to improve the performance of PEMFC and their components resulted in the necessity of using Nafion membranes of <100 µm thickness. Reducing the thickness of the electrolyte reduces the system resistance and increases the power of the PEMFC [8]. Membranes of this thickness can be obtained by casting from polymer dispersions and/or solutions [5]. Nafion^®^ 212 and Nafion^®^ 211 membranes with thicknesses of 50 and 25 µm, respectively, are commercially available today. The influence of the thickness of Nafion^®^ samples, obtained by casting and extrusion (commercial and laboratory fabricated), on their resistivity was investigated [9,10,11,12,13]. While the areal resistivity (Ohm∙cm^2^) increases as the thickness of the sample increases, the specific resistivity (Ohm∙cm) decreases by contrast [9]. When the charge carrier crosses the membrane/electrode interface, an additional resistance arises, the contribution of which for perfluorosulfonic membranes with low internal resistance is rather significant and is higher the thinner the membrane is.

For some time, the problem of low mechanical strength of Nafion-type membranes obtained by casting was an issue, but this can be overcome by an optimal solvent removal temperature regime [14]. In most solvents, perfluorosulfonic polymers form dispersions because of the incompatibility of the liquid and the hydrophobic main chain of the polymer [15]. The morphology of the polymer in the dispersion and in the membrane changes depending on the nature of the dispersing liquid and the counterion. A number of papers show that the microstructure of the Nafion polymer in the dispersion strongly depends on the composition and conditions of its formation [15,16,17,18,19]. Since in such systems the polymer is already structured in a certain way, this also affects the properties of the obtained membranes. During solvent removal from Nafion^®^ dispersions, gelation occurs by different mechanisms, depending on the nature of the solvent [20]. The mechanical properties of casting membranes differ by up to four orders of magnitude depending on the dispersing liquid. The membranes obtained from dispersions in aprotic solvents are the most durable. It has been suggested that the mechanical strength of membranes is higher the more entangled the polymer chains are with each other during the removal of the dispersing liquid [20]. Ionic conductivity of perfluorosulfonic acid recast membranes also varies greatly depending on the nature of the solvent [21,22]. A large number of studies have been devoted to the morphology of Nafion-type membranes [7,23,24,25]. In the past decade, the appearance of a large number of works devoted to the description of the morphology of perfluorosulfonic acid membranes in dispersions and solutions can be noted [15,19,26,27,28]. Mixtures based on water and low-molecular-weight alcohols, aprotic solvents or glycols are used as dispersing liquids [27,28,29,30,31]. There are few works devoted to the study of the relationship between the morphology of perfluorosulfonic acid polymers in the dispersion and in the membrane film.

Upon forming PEMFC, perfluorosulfonic acid polymers are used not only as an electrolyte, but also as a binder and proton conducting component to create catalytic inks for membrane-electrode assembly (MEA) of PEMFC [32]. The composition and conditions of catalyst ink pretreatment have a significant influence on the performance and lifetime of the PEMFC [33,34,35]. The thin layer of perfluorosulfonic acid polymers on the surface of the catalyst nanoparticles ensures rapid proton transfer and makes a significant contribution to the efficiency of the catalytic layer in the PEMFC [35,36]. The study of the relationship between the morphology of Nafion^®^ perfluorosulfonic acid polymers in dispersions and thin films (several tens of nanometers thick) makes it possible to identify the causes of degradation of MEAs components and determine approaches to optimize their properties [19,37,38]. The morphology of polymer thin films determines their proton conductivity and affects the rate of water transfer. The change in dispersion composition also affects the aggregation of the catalyst and polymer; the interaction between the ionomer and catalyst particles during MEA formation [26,29,39,40]. Thus, the proper choice of solvent for the dispersion can improve the characteristics of the catalytic layer and PEMFC.

Most of the earlier published works were devoted to the investigation of the membranes’ casting conditions using various dispersions, but do not discuss the difference in such properties of the obtained membranes as ionic conductivity and water uptake, which are the most important electrolyte characteristics in PEMFC. Thus, the aim of this work is to compare the properties of Nafion dispersions in various dispersing liquids and the properties of the membranes recast from them. Aprotic solvents such as *N,N*-dimethylformamide (DMF), *N*-methyl-2-pyrrolidone (NMP) as well as isopropyl alcohol–water (IPA-H_2_O) mixtures in various volume ratios and ethylene glycol (EG) were used as dispersing liquids. The choice of dispersing liquids was due to the difference in the affinity of the polymer and boiling point and is based on the information presented in the literature about the possibility of dispersing Nafion polymers in them.

## 2. Materials and Methods

### 2.1. Materials

The following materials and reagents were used: Nafion^®^ 212 membrane (equivalent weight 1100, The Chemours Company, Wilmington, DE, USA), *N*-methyl-2-pyrrolidone (Merck, Darmstadt, Germany), *N,N*-dimethylformamide (special purity, Chimmed, Moscow, Russia), ethylene glycol (special purity, Chimmed, Moscow, Russia), isopropyl alcohol (special purity, Chimmed, Moscow, Russia), lithium hydroxide (LiOH, ≥99.0%, Sigma-Aldrich, St. Louis, MO, USA), hydrochloric acid (special purity, 35–38%, Chimmed, Moscow, Russia), potassium chloride (reagent grade, Chimmed, Moscow, Russia), potassium hydroxide (reagent grade, Chimmed, Moscow, Russia); deionized water (resistance 18.2 MOhm).

### 2.2. Obtaining Dispersions

Perfluorosulfonic acid polymers dispersions were obtained from Nafion^®^ 212 membranes in different ionic forms, which was preliminarily conditioned under standard conditions [41]. The conversion into Li^+^, Na^+^, and Ca^2+^ forms was performed by soaking membranes in a 1 M solution of LiOH, NaOH or CaCl_2_ with a 3-fold replacement of the solution with a new one every 2 h. The obtained membranes were washed with deionized water. The use of the polymer in these cationic forms was chosen for two reasons: (i) the increase in its thermal stability when transferred to alkali or alkaline earth metal cations in comparison to protons, which will allow obtaining dispersions and forming membranes in a wider temperature range; (ii) the influence of counterions on the viscosity of the polymer dispersion. The completed conversion of the membrane to the appropriate ionic form was controlled by acid–base titration, the methodology of which is described below. To obtain the dispersions, the membrane sample in the appropriate ionic form was pre-dried in an OV-11/12 vacuum oven (Jeio tech, Daejion, Korea) at 50 °C for 6 h. The film suspension was ground and placed in a round-bottom flask or solvothermal cell together with the dispersing liquid to obtain 5 wt.% dispersion.

The conditions for obtaining dispersions were experimentally selected depending on the nature of the dispersing liquid (temperature, time of thermal treatment, type of counterion). The temperature of obtaining dispersions was gradually increased to determine the minimum at which the polymer was completely dispersed, and then the processing time was gradually reduced to determine the minimum required. As a result, dispersions of Nafion were obtained in the following solvents: *N,N*-dimethylformamide (DMF), *N*-methyl-2-pyrrolidone (NMP), ethylene glycol (EG) and a mixture of isopropyl alcohol (IPA) with water in various ratios (IPA-H_2_O, V:V = 80–20, 60–40, 40–60, 20–80). In pure isopropyl alcohol and water, the polymer does not disperse even when treated at high temperature up to 230 °C in solvothermal conditions. The results of selection of optimal conditions for obtaining dispersions are presented in Table 1.

### 2.3. Membrane Formation by Casting Method

To form the membranes by casting procedure, the obtained dispersions were poured onto the surface of a glass Petri dish and heated to remove the solvent. The optimal drying conditions for complete solvent removal were selected experimentally (Table 1). The mass of the membrane after solvent removal was 4.9–5.0 wt.% of the solution mass. The density of the films in the dry state was 1.5–1.7 g/cm^3^. To cast the membranes with the required thickness, the necessary volume of solution was calculated from the volume of the recast membrane, its density and the concentration of the polymer in the dispersion. Preliminary experiments were performed to evaluate the density of the membrane. The obtained films were removed from the glass surface and subjected to hot pressing at a pressure of 5 MPa at 110 °C for 3 min to increase the strength. The films were conditioned to bring them to standard conditions and transfer them into proton form. For this purpose, they were successively treated at room temperature first, twice with a 5 wt.% HCl solution for 1.5 h and then washed with deionized water before the Cl^−^ ions reaction disappeared. The presence of Cl^-^ ions in the solution was controlled by the reaction with AgNO_3_. The samples were stored in deionized water. The properties of the H^+^-form membranes were studied.

Thus, the following membranes were obtained from polymer dispersions in the Li^+^-form: M[IPA-H_2_O 80–20], M[DMF], M[NMP] and M[EG].

### 2.4. Methods

The density of dispersing liquids and dispersions was determined using a Densito portable density meter (Mettler Toledo, Greifensee, Switzerland) at 25 ± 0.1 °C. Viscosity was determined using a vibrating viscometer SV-1A (A&D, Tokyo, Japan) at 25 ± 0.2 °C. The value of dynamic viscosity (η, mPa∙s) was calculated from the ratio of the experimentally obtained viscosity to the liquid density. The viscometer was calibrated by two points using 5 and 10 mPa∙s viscosity standards (Brookfield, Toronto, ON, Canada). Viscosity was calculated as an average of three independent experiments.

^19^F NMR spectra of Nafion dispersions were collected using an AVANCE II 600 NMR spectrometer (Bruker, Billerica, MA, USA).

The IR spectra of the membranes in a dry state were recorded on a Nicolet iS5 FTIR spectrometer (Thermo Fisher Scientific, Waltham, MA, USA) with the Fourier transformation and ATR add-on (a diamond crystal).

The ion-exchange capacity of the membranes (IEC, mg-eq/g) was determined by direct acid–base titration using an Expert 001 pH-meter (Econix-Expert, Moscow, Russia). Membranes in H^+^-form were preliminarily kept at 150 °C for 30 min to remove water. The sample weight of ~0.3 g was stored in 50 mL of 0.1 M NaCl solution for 6 h under constant stirring. After that, the membrane was decanted from salt solution. The salt solution was titrated with 0.05 M NaOH solution. The IEC was calculated relative to the weight of the dry membrane in H^+^-form.

The mechanical properties of the membranes, i.e., break stress and strain, Young’s modulus and proportional limit stress were examined using an H5KT tensile test machine (Tinius Olsen, Horsham, UK) with a 100 N force transducer at 25 ± 2 °C and relative humidity RH = 20 ± 2%. The tensile rate of uniaxial deformation was 5 mm/min. The films were held at a relative humidity of RH = 32% beforehand in a desiccator under saturated CaCl_2_ solution. Rectangular samples with a length of 70 mm (gauge length was 45 mm) and a width of 7 mm were used. Five experiments were performed for each membrane. Thickness and width were determined immediately before the experiment as an average of 5 points along the entire length (using a Mitutoyo micrometer, 0.001 mm accuracy). Young’s modulus was determined from the slope of the stress–strain curve in the elastic strain region. Mean values were calculated for each series of samples, and the error of measurement was evaluated by Student’s t-distribution.

Water uptake of the membranes pre-equilibrated in contact with water or at 95% and 30% relative humidity for 24 h was determined using TG 209 F1 (Netzsch, Selb, Germany). The amount of water in the sample was calculated as the ratio of the mass difference before heat treatment and after holding at 150 °C for 30 min, to the mass of the sample before heat treatment.

Proton conductivity of the membranes was studied at the RH = 100% in contact with deionized water in the temperature range 25–85 °C and at RH = 95% and 30% in the temperature range 25–60 °C. A climate chamber of constant conditions MKF115 (Binder, Tuttlingen, Germany) was used to set the necessary humidity (humidity setting accuracy ± 2.5%) and temperature. Measurements were performed using an AC bridge E-1500 (Elins, Chernogolovka, Russia) in the frequency range of 10 Hz to 3 MHz on symmetrical carbon/membrane/carbon cells with an active electrodes surface area of 1 cm^2^. The thickness of the membranes was determined after the equilibration at the given humidity for each experiment using a Mitutoyo micrometer (0.001 mm accuracy). The value of conductivity (Ohm^−1^∙cm^−1^) was calculated from the resistance found from the impedance hodograph at the cutoff along the axis of active resistance. The error in determining the value of conductivity was less than 10%.

Cation transport numbers across membranes in the Na^+^-form were determined at 23 ± 1 °C using the procedure described in [43]. To convert the membranes into Na^+^-form, they were soaked for 24 h in 2 M NaCl and washed repeatedly with deionized water. Before the experiment, the membrane samples were kept for 12 h in 0.1 M NaCl solution. After that, the sample was placed in a two-chamber cell separated by a membrane (the volume of each chamber was 32 cm^3^). The concentrations of NaCl solutions were 0.1 M in one chamber and 0.5 M in the other. Two Luggin capillaries and Ag-AgCl electrodes were placed in each chamber. The membrane potential E_mes_ between the electrodes was measured using a potentiostat-galvanostat P-8 nano (Elins, Chernogolovka, Russia). The membrane potential was calculated according to the equation E_mem_ = E_mes_ − E_corr_, where E_mes_ is the measured value, E_corr_ is the value taking into account the non-ideality of reference electrodes, which was determined using a Neosepta CMX© membrane as the internal standard (transport number under the studied conditions––99.0% [44]). The cation transport numbers ($t_{+app}$) were calculated as the ratio of the found potential to the potential of the ideally selective membrane by the Equation:

(1) $$t_{+app}=\frac{E_{mem}}{E_{max}\frac{RT}{F}ln{\left(\frac{a_{1}}{a_{2}}\right)}_{max}}$$

where R is the gas constant, T is the absolute temperature, K, F is Faraday’s constant, $a_{1}$ and $a_{2}$ are the electrolyte activities (the values were found by interpolation by continuous function of the table values ($a_{1}$ (0.5 M NaCl) = 0.339, $a_{2}$ (0.1 M NaCl) = 0.0773)). The error of determination of $t_{+app}$ was ±0.2%.

Hydrogen permeability through the membranes was determined by gas chromatography using a Crystallux-4000 M chromatograph (NPF Meta-Chrom, LLC, Mari El Republic, Yoshkar-Ola, Russia) with a thermal conductivity detector (current 30 mA) and a packed column (Mole Seive sorbent 5 Å, 2 m, 30 °C, 20 cm^3^/min, Ar) at RH = 95% humidity. To produce hydrogen, a pure hydrogen generator GVCh-12A (Khimelektronika Ltd., Moscow, Russia) was used. The experiment was carried out in a thermostatted two-chamber cell with an active surface area of 4 cm^2^. Pure hydrogen was fed into one chamber, and argon was fed into the other chamber at the rate of 20 mL/min. To create the necessary level of humidity in the flow of hydrogen and argon, each gas was passed through two bubbler tanks with water, which were thermostatted at the same temperature as the cell. The hydrogen permeability coefficient P (cm^2^/s) was calculated using the Equation:

(2) $$P=\frac{jL}{C_{H}-C_{Ar}}$$

where L is the membrane thickness, $C_{H}$ is the average volume concentration of hydrogen in the chamber in which hydrogen was supplied, $C_{Ar}$ is the average volume concentration of hydrogen in the chamber in which argon was supplied. The gas flow through the membrane j was calculated from the Equation:

(3) $$j=\frac{C_{Ar}V_{t}}{S}$$

where $V_{t}$ is the volumetric velocity of the carrier gas flow, S is the active area of the membrane.

## 3. Results and Discussions

### 3.1. Properties of Nafion Dispersions

**Figure 1:**
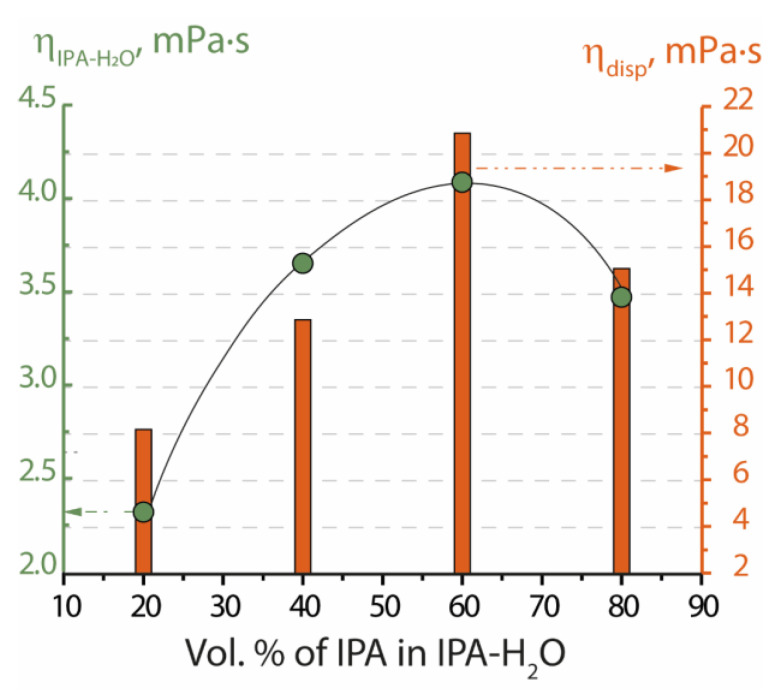
Dependences of solvent viscosity (dots) and viscosity of 5 wt.% Nafion dispersions in Li^+^-form (columns) on the IPA volume fraction in the mixture IPA-H_2_O.

When dispersing perfluorosulfonic acid polymers in an IPA-H_2_O mixture, the conditions for obtaining dispersions and their properties vary depending on the volume fraction of water (Table 1). In pure isopropyl alcohol, the polymer does not disperse even when treated at a high temperature up to 230 °C in solvothermal conditions. At this temperature, the processes of chemical degradation of the polymer initiate [7]. Nafion dispersion in pure isopropyl alcohol can be obtained only by replacing water from the IPA-H_2_O dispersion, but according to small-angle X-ray scattering data, such a system contains large spherical polymer particles with a low solvent fraction [15]. Nafion perfluorosulfonic acid polymers are dispersed most easily in mixtures with high isopropyl alcohol content (IPA-H_2_O 60–40 and IPA-H_2_O 80–20). A short treatment at 80 °C with constant stirring is sufficient to disperse the polymer (Table 1). However, in mixtures with low isopropyl alcohol content (IPA-H_2_O 20–80 and IPA-H_2_O 40–60), the dispersion can only be obtained by treatment in a solvothermal cell at 150 °C. The dependences of dispersion and solvent viscosities on the volume fraction of isopropyl alcohol in the mixture have a similar pattern and pass through a maximum at 60 vol.% isopropyl alcohol (Figure 1). When a small amount of water is added to the isopropyl alcohol, associates (H_2_O)_n_ are formed, surrounded predominantly by OH groups of the alcohol. However, once a certain ratio of the number of IPA-H_2_O molecules is reached, further increasing the water fraction leads to a decrease in the viscosity of the mixtures, since the water molecules form a three-dimensional network, which is disturbed by the inclusion of alcohol molecules or associates. The same effect takes place for polymer dispersions. The change in viscosity is also associated with a change in polymer morphology at different alcohol percentages. For example, the morphology of Nafion in an ethanol–water dispersion was studied in the work [28], where at an ethanol content of <50 wt.%, the polymer was presented by the cylinders, while at a higher ethanol content Nafion acquired the conformation of random coils. This is explained by the strong electrostatic interactions between the charged groups, whereas the greater interfacial attraction of the polymer/solvent and greater conformational entropy of PFSA chains at high concentrations of ethanol results in a highly solvated structure.

Nafion polymer disperses well in aprotic solvents *N*-methyl-2-pyrrolidone (at 100 °C) and *N,N*-dimethylformamide (at 120 °C) (Table 1). Ethylene glycol disperses the polymer only at 180 °C and forms a dispersion with high viscosity η = 35.1 mPa∙s. The viscosity of Nafion dispersions in Li^+^-form increases from 5.5 to 35.1 mPa∙s in the series DMF < NMP < IPA-H_2_O 80–20 < EG.

The choice of the ionic form of the Nafion polymer is also important for the formation of dispersions. It was possible to obtain a dispersion of the Nafion in Ca^2+^-form in *N,N*-dimethylformamide only when the temperature was increased up to 200 °C. A brown liquid was formed. The brownish color indicates the formation of anhydrides by condensation of two -SO_3_^−^-groups of Nafion polymer under high temperatures [45]. Thus, a recast of membranes from such dispersions is meaningless. Differences in the temperature of obtaining dispersions of Nafion in Li^+^ and Ca^2+^ forms in *N,N*-dimethylformamide are caused only by the difference in the strength of the electrostatic interaction between the functional groups inside the pores. The interaction of two-charged cations with sulfo-groups limits the mobility of the polymer side chains and prevents its dispersion. This can be compared to an increase in the number of “cross-links” between the chains, which prevents their solubility. The viscosity of the resulting dispersion was almost three times higher than that of the polymer dispersion in the Li^+^ form (η = 14.5 and 5.5 mPa∙s for the Ca^2+^ and Li^+^ form, respectively, Table 1).

The viscosity of polyelectrolyte solutions is greatly influenced by the molecular weight, the shape of the polymer macromolecules and the polymer–solvent interaction. Since the molecular weight of all polymers is the same, viscosity changes are related to the polymer affinity to the solvent and the polymer morphology in dispersion, which is determined by polymer–polymer and polymer–solvent interactions. Viscosities of polymer dispersions in Li^+^ and Na^+^ ionic forms obtained under the same conditions in the IPA-H_2_O 80–20 mixture were close. At the same time, the viscosity of the polymer dispersion in H^+^-form was 1.6 times higher (Table 1). The reason for this seems to be the formation of intermolecular hydrogen bonds.

The data of small-angle X-ray neutron scattering and atomistic molecular dynamics simulation showed that the size of Nafion agglomerates and their shape in contact with different liquids vary greatly [15,27]. According to these studies, in an aqueous-alcohol dispersion, the polymer is organized as highly solvated large particles of about 200 nm in size with a high degree of agglomeration. At the same time, in contact with aprotic solvents such as *N*-methyl-2-pyrrolidone and *N,N*-dimethylformamide, the morphology of Nafion is closest to the real solution [15]. In aprotic solvents, polymer macromolecules are not aggregated and represent the conformation of chaotic random coils of several nanometers in size. This is due to the high affinity of the polymer chains to the dispersing liquid.

**Figure 2:**
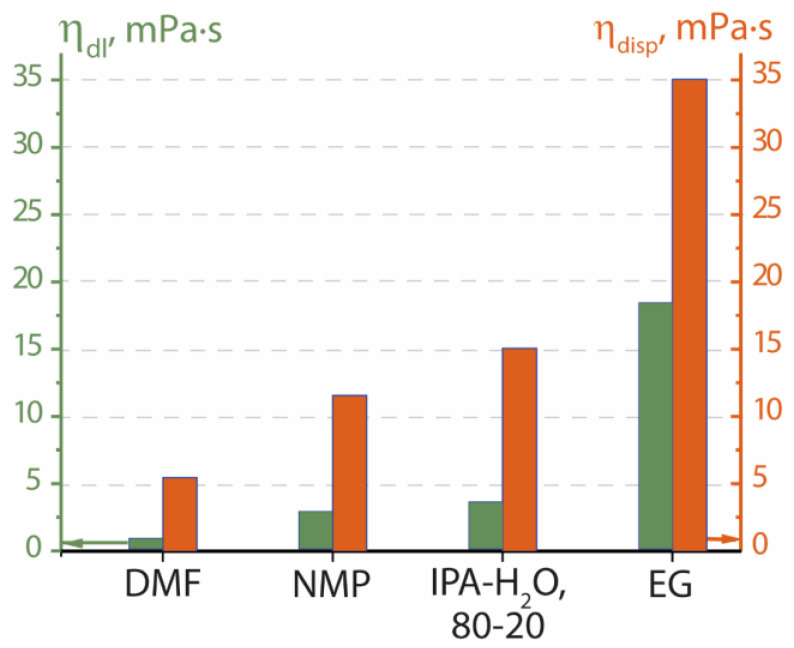
Viscosity of dispersing liquids (η_dl_) and dispersions of Nafion (5 wt.%) in Li^+^-form (η_disp_) in different liquids.

When the polymer and dispersing liquid were mixed, the viscosity of the dispersion increased significantly (Figure 2). Thus, the absolute increase in viscosity of dispersions in *N,N*-dimethylformamide and *N*-methyl-2-pyrrolidone was relatively small, which is tied in with the comparably small interaction of individual components. The viscosity of ethylene glycol and Nafion dispersion in ethylene glycol was the highest among the liquids studied (Table 1, Figure 2). The increase of the viscosity in this case was also maximum. According to the data of small-angle X-ray neutron scattering, in the presence of ethylene glycol, macromolecules form cylinders of small diameter (about 5 nm) [15]. The connectivity of such chains when they move relative to each other is noticeably greater.

**Figure 3:**
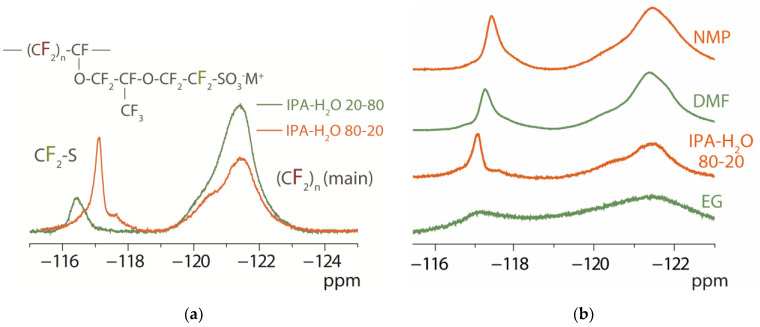
The fragments of ^19^F NMR spectra of dispersions of Nafion in Li^+^-form (5 wt.%) in IPA-H_2_O mixtures in different ratios (**a**) and NMP, DMF, IPA-H_2_O 80-20, and EG (**b**) dispersing liquids.

Another important parameter for the formation of the membrane with optimal morphology is the mobility of the side chains in the dispersion. The mobility of the side chains of perfluorosulfonic acid polymer in 5 wt.% Nafion dispersions was estimated from ^19^F NMR spectroscopy data by the line width of the CF_2_ group of the side chain closest to the functional sulfo-group (Figure 3). The peak at ~−121.5 ppm corresponded to the CF_2_ groups of the main chain and its position was independent of the dispersion composition. The peak in the ~−117 ppm region corresponded to the CF_2_ group of the side chain closest to the functional sulfo-group [15,46]. Its position varied depending on the surroundings of the polymer. In the IPA-H_2_O mixture, the position of this signal was shifted to the low field compared to aprotic solvents such as *N*-methyl-2-pyrrolidone and *N,N*-dimethylformamide. This is due to the fact that dissociation of sulfo-groups occurs in the presence of water, which leads to an increase in the electron density on the fluorine atoms near them.

The signal width at −117 ppm in the ^19^F NMR spectra of the polymer dispersions decreased in the series EG ≪ IPA-H_2_O 20–80 < DMF ~ NMP < IPA-H_2_O 80–20 (Figure 3). This could be a consequence of increased viscosity of the dispersions. However, the viscosity decreased in the series EG ≫ IPA-H_2_O 80–20 > NMP > IPA-H_2_O 20–80 > DMF (Table 1). A low degree of macromolecules’ agglomeration and no electrostatic interaction of polymer in contact with aprotic solvents *N,N*-dimethylformamide and *N*-methyl-2-pyrrolidone provide high mobility of side chains. Increasing the water fraction in the IPA-H_2_O system led to a decrease in mobility of side chains (Figure 3a). The side chain mobility of the IPA-H_2_O 20–80 dispersion was significantly lower despite the lower viscosity of the IPA-H_2_O 20–80 dispersion compared to the IPA-H_2_O 80–20 dispersion (Table 1). Thus, it is shown that the nature of the dispersing liquid and the type of counterion affect the viscosity of Nafion dispersions, the morphology of the polymer in them and the mobility of the side chains.

### 3.2. Membrane Properties

The morphology of the polymer in the dispersion and the dissociation degree of the functional groups affect the process of pore and channel system formation and the ability of the hydrophobic polymer matrix to undergo deformation due to water sorption by sulfo-groups during hydration. The main perfluorinated chains of Nafion are hydrophobic, while the side chains with sulfo-groups at the ends are hydrophilic (see Nafion polymer structure in Figure 3a). In the process of solvent removal, the ion exchange groups aggregate into clusters with a hydrophobic matrix around them. The formation of stable films from IPA-H_2_O dispersions with water content ≥ 40 vol.% is not possible. With increasing water content in the dispersing liquid, micelles are formed, in which the functional groups predominantly emerge on the surface, which prevents the entanglement of macromolecules and does not allow films with sufficient mechanical properties to be obtained. Because of the slight difference in the viscosity of Nafion dispersions in the Li^+^ and Na^+^-forms in *N,N*-dimethylformamide (Table 1), all membranes were obtained from a polymer in Li^+^ form. The properties of the following membranes will be described below (the dispersing liquid is indicated in square brackets): M[IPA-H_2_O 80–20], M[DMF], M[NMP], and M[EG].

**Figure 4:**
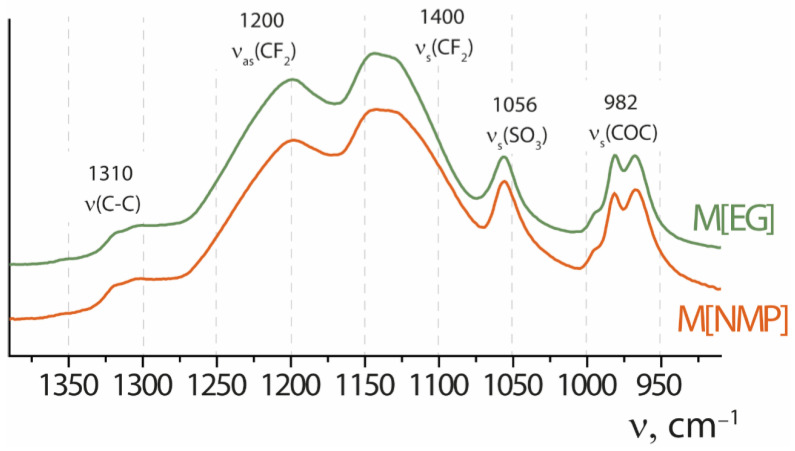
Fragments of IR spectra of some membranes in H^+^-form: M[DMF] and M[EG]. The samples were pre-dried at 50 °C under vacuum for 24 h.

The IEC of the obtained membranes is close to the initial Nafion^®^ 212 (0.95–0.96 mg-eq/g), from which the dispersions were recast. The IEC of the M[DMF], M[IPA-H_2_O 80–20] and M[NMP] membranes was 0.94–0.96 mg-eq/g. The lowest IEC value was obtained for the M[EG] membrane (0.91 mg-eq/g). Some of the decrease compared to the Nafion^®^ 212 membrane can be attributed to a change in the availability of functional groups and/or a slight difference in the amount of water retained by the sample. This difference is most pronounced for membranes obtained from dispersion in ethylene glycol, in which these processes are revealed by a high processing temperature (180 °C) and possibly accompanied by some degradation of the polymer. At the same time, according to IR spectroscopy data, there were no changes in the position and intensity of vibrations of the main groups of the Nafion polymer as a result of dispersion and subsequent casting (Figure 4).

The mechanical properties of the membranes were studied (Table 2). The M[EG] membrane has low strength and breaks down under low stress in the region of reversible deformations. The mechanical properties of polymers are determined by the degree of entanglement of the macromolecules [20]. According to the assumption that in the dispersion in ethylene glycol, Nafion already represents cylinders of small size [15], it can be assumed that macromolecules are not significantly rearranged when the solvent is removed and the film is formed. The low cohesion of the primary structures leads to unsatisfactory mechanical properties. In the series M[IPA-H_2_O 80–20] < M[NMP] < M[DMF] the Young’s modulus increases 4 times and the proportional limit stress increases almost twice (Table 2). Young’s modulus characterizes the rigidity of membranes and in the case of perfluorosulfonic acid polymers is determined by the strength of electrostatic interactions within pores [47]. The higher value of Young’s modulus for M[DMF] membranes as compared to M[NMP] may be related to the size of the pores formed and the number of functional groups in them.

The tensile strength and strain values differ greatly for each sample (Table 2), which is typical for Nafion-type membranes [47]. However, it can be noted that the M[NMP] samples have the highest strength and strain capability. The morphology of small-sized random coils, which is typical for Nafion in aprotic solvents [15], as well as the low viscosity of dispersions contribute to the efficient entanglement of macromolecules and the formation of a hydrophobic matrix with fine mechanical properties. The slower rate of solvent removal in the case of dispersion in *N*-methyl-2-pyrrolidone compared to the dispersion in *N,N*-dimethylformamide contributes to additional binding of macromolecules and increases their strength and deformation ability. When obtaining M[IPA-H_2_O 80–20] membranes from dispersions in which the polymer is in the form of large agglomerates [15], the mechanical properties are significantly inferior to those of membranes obtained from aprotic solvents because of the lower degree of entanglement of polymer chains. The presence of water in the system leads to dissociation of sulfo-groups and formation of micelles with a hydrophilic surface, which hinders the contact of hydrophobic chains forming the matrix. This leads to a decrease in mechanical strength. In general, it should be noted that the mechanical properties of all the studied materials, except for the samples obtained from dispersion in ethylene glycol, allow their use as an electrolyte in PEMFC.

The water uptake of the obtained membranes also significantly depends on the composition of the dispersion from which they are recast and decreases with decreasing water activity in the medium (Table 3). In contact with water, the water uptake of the membranes obtained from aprotic solvents and the water–alcohol mixture was close and amounted to about 24.9–25.2 wt.%. At RH = 95% and 32%, M[DMF] were characterized by the highest water uptake. Lower water uptake was observed for M[IPA-H_2_O 80–20] and M[NMP] membranes. The minimum water uptake was obtained for M[EG] samples.

The water uptake of perfluorosulfonic acid membranes depends on the number of functional groups (IEC of membranes) [7]. The change in the membrane water uptake depending on the composition of the dispersions from which it is obtained correlated well with their IEC. The lowest water uptake was obtained for the membranes in which a part of the sulfo-groups was lost during heat treatment (M[EG]). At the same time, it can be noted that the difference in the IEC of M[DMF] and M[EG] membranes was about 5%, and the difference in water uptake reaches 30–40%. The second factor determining the water uptake of membranes is the possibility of pore expansion during their hydration. It depends mainly on mobility of the polymer perfluorinated chains located close to pores. A significant difference in the water uptake of M[DMF] and M[EG] membranes is due to the difference in their microstructure. The Nafion polymer in ethylene glycol represents cylinders in which walls are formed from perfluorinated chains [15]. Thus, in M[EG] membranes, deformation of the polymer matrix is limited and sorption of a large amount of water is impossible. This leads to a low water uptake of the membranes.

**Figure 5:**
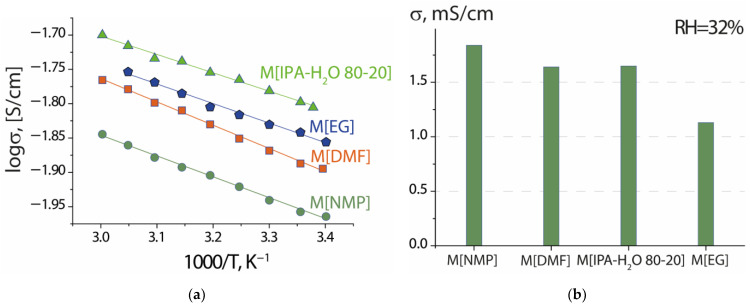
Temperature dependence of proton conductivity measured in contact with water (**a**) and at 25 °C and RH = 32% (**b**) of the studied membranes.

The proton conductivity of the membranes in contact with water and at RH = 32% was studied (Figure 5). The conductivity of all membranes was comparable and varied from 15.5∙10^−3^ to 22.3∙10^−3^ Ohm^−1^∙cm^−1^ at t = 30 °C in contact with water and from 11.3∙10^−4^ to 20.4∙10^−4^ Ohm^−1^∙cm^−1^ at t = 25 °C and RH = 32%. In contact with water, the conductivity increased in the series: M[NMP] < M[DMF] < M[EG] < M[IPA-H_2_O 80–20]. In general, conductivity values were in good agreement with changes in membrane water uptake except for M[EG] (Table 3). A non-trivial fact is the rather high conductivity of the sample obtained from the dispersion in ethylene glycol, given the low values of its IEC and water uptake (Table 3). It can be assumed that the reason for its high proton conductivity is the possibility of long-distance proton transport along the cylinder surface where the sulfo-groups are located. From this point of view, it cannot be excluded that this membrane may possess anisotropy of sorption and transport properties. A similar effect was observed when the ion transport rate increased in Nafion-type membranes after their mechanical deformation by stretching [47].

Conductivity of the studied samples at RH = 32% was more than an order of magnitude lower than in contact with water and increased in the series M[EG] < M[DMF] ~ M[IPA H_2_O 80–20] < M[NMP] (Figure 5b). Under these conditions, the conductivity of the sample M[EG], as well as its water uptake, had the minimum value among the studied samples. This is apparently a result of the fact that part of the functional groups is located on the surface of the micelles and takes part in the process of fast proton transfer. On the other hand, this also contributes to their easy dehydration, which determines the rapid decline in conductivity with decreasing humidity.

The transfer of cations in Nafion membranes is carried out mainly within the thin Debye layer near the membrane pore walls, while the transfer of anions and non-polar molecules is carried out through the electroneutral solution inside the pores [48,49]. The cation transport selectivity and gas permeability of Nafion membranes are important characteristics both in terms of their application and in terms of understanding the microstructure. These characteristics were studied for all the investigated membranes except for M[EG], since it was not possible to obtain a qualitative sample of the size required for such experiments. The cation transport numbers of membranes in the Na^+^-form ranged from 83% to 85% (Table 4). The samples obtained from aprotic solvents had slightly higher values than samples obtained from an IPA-H_2_O mixture. The hydrogen permeability through the membranes obtained from aprotic solvents is comparable with that of the Nafion^®^ 212 membrane ((1.34 ± 0.06)∙10^−7^ cm^2^/s at RH = 95% and t = 25 °C) [50] and 1.5 times lower than for M[IPA-H_2_O 80–20] (Table 4).

The selectivity and gas permeability of ion-exchange membranes usually correlate with the change in the volume of the electroneutral solution within the pore: the larger it is (i.e., the higher the water uptake), the higher the rate of non-selective transport is. In contact with water, the water uptake of M[NMP] and M[DMF] membranes was the same or higher than M[IPA-H_2_O 80–20] (Table 3). Thus, the same or higher gas permeability values for the first group of membranes is expected. However, for membranes made from aprotic solvents, gas permeability was 1.5 times lower (Table 4). The degree of Nafion polymer agglomeration in *N,N*-dimethylformamide and *N*-methyl-2-pyrrolidone is low [15] and membranes with a more branched pore and channel system with a uniform size distribution are formed when the solvent is removed from such dispersion. The rate of non-selective transport through such membranes is lower. As a result, PEMFCs based on membranes recast from dispersions in aprotic solvents have higher capacity as compared to the Nafion^®^ 212 membrane by up to 27% [50].

## 4. Conclusions

Dispersions of the perfluorosulfonic acid polymer Nafion in various dispersing liquids (water–isopropyl alcohol mixtures in different ratios, the aprotic solvents (*N,N*-dimethylformamide and *N*-methyl-2-pyrrolidone), and ethylene glycol) and with different counterions were studied. The viscosity of the dispersions in Li^+^-form increased in the series DMF < NMP < IPA-H_2_O 80–20 < EG from 5.5 to 35.1 mPa∙s. According to ^19^F NMR spectroscopy data, the mobility of side chains of dispersions of Nafion in Li^+^-form increased in the series EG ≪ IPA-H_2_O 20–80 < DMF ~ NMP < IPA-H_2_O 80–20.

It was shown that the morphology of the polymer in the dispersion determines the mechanical properties of the membranes formed by casting from Nafion dispersions in Li^+^-form. Membranes obtained from dispersions in aprotic solvents had the highest strength and rigidity. The high mobility of macromolecules in dispersions in aprotic solvents and the low degree of their agglomeration contributed to the effective bonding of macromolecules and the formation of films with good mechanical properties.

M[IPA-H_2_O 80–20], M[DMF] and M[NMP] membranes have high values of water uptake and proton conductivity. Casting the membranes from dispersions in aprotic solvents led to the formation of uniform pores of small size with a good interconnectivity. As a result, the rate of non-selective transport through such membranes was lower than through M[IPA-H_2_O 80–20].

## Figures and Tables

**Table 1:** Conditions for obtaining dispersions, viscosity (η, mPa∙s at 25 °C) of dispersing liquids (η_dl_) and dispersions (η_disp_) containing 5 wt.%, Nafion polymer.

Dispersing Liquid	Boiling Point, °C	η_dl_, mPa·s	Counterion	Conditions for Obtaining the Dispersion **	η_disp_., mPa·s	Membrane Formation Conditions	Membrane Description ***
IPA-H_2_O 100–0	82.2 *	2.1	H^+^, Li^+^	Does not disperse when processed in the STC up to 230 °C 6 h	-	-	-
IPA-H_2_O 80–20	80.8 *	3.5	H^+^	80 °C in RBF with constant stirring for 2 h	24.6	40 °C 3 h in air then 80 °C 3 h in vacuum oven	Mechanically stable, transparent membrane, *l* > 70 µm
			Li^+^		15.1		
			Na^+^		17.4		
IPA-H_2_O 60–40	81.0 *	4.1	Li^+^	80 °C in RBF with constant stirring for 2 h	20.9	40 °C 3 h in air, then 80 °C 3 h in vacuum oven	Poor mechanical properties, it is possible to obtain samples of small size
IPA-H_2_O 40–60	81.4 *	3.7	Li^+^	150 °C in STC for 3 h	12.9	40 °C 3 h in air, then 80 °C 3 h in vacuum oven	The membrane does not form
IPA-H_2_O 20–80	82.6 *	2.3	Li^+^	150 °C in STC for 3 h	8.2	40 °C 3 h in air, then 80 °C 3 h in vacuum oven	The membrane forms
DMF	153	0.8	Li^+^	120 °C in STC for 2 h	5.5	40 °C 3 h in air, 60 °C 3 h in air, 120 °C 6 h in vacuum oven	Mechanically stable, transparent membrane, *l* > 70 µm
			Na^+^	120 °C in STC for 2 h	5.7	40 °C 3 h in air, 60 °C 3 h in air, 120 °C 6 h in vacuum oven	Mechanically stable, transparent membrane, *l* > 70 µm
			Ca^2+^	200 °C in STC for 12 h	14.5	-	-
EG	195–198	18.3	Li^+^	180 °C in STC for 6 h	35.1	140 °C 6 h in a vacuum oven	Transparent brown membrane, *l* > 100 µm
NMP	202	2.8	Li^+^	100 °C in STC for 2 h	11.6	60 °C 3 h in air, 120 °C 6 h in vacuum	Transparent membrane, *l* > 50 µm

* Data from [42]. ** STC –solvothermal cell, RBF–round bottom flask; *** *l*–dry thickness

**Table 2:** Mechanical properties of membranes in H^+^-form kept at RH = 32%.

Membrane	Young’s Modulus, MPa	Proportional Limit Stress, MPa	Tensile Strength, MPa	Tensile Strain, %
M[IPA-H_2_O 80–20]	228 ± 20	6.7 ± 1.3	7.7 ± 1.4	11 ± 2
M[NMP]	268 ± 10	11.6 ± 0.4	19.8 ± 0.6	241 ± 11
M[DMF]	319 ± 14	12.9 ± 0.5	17.0 ± 0.6	102 ± 5

**Table 3:** Water uptake (wt.%) of the studied membranes in H^+^-form at different relative humidity.

Membrane	In Contact with Water, %	RH = 95%, %	RH = 32%, %
M[IPA-H_2_O 80–20]	25.1	17.6	4.8
M[DMF]	25.2	19.5	5.6
M[NMP]	24.9	16.6	4.8
M[EG]	15.3	11.7	4.0

**Table 4:** Cation transport numbers ($t_{Na^{+}}^{+}$, %) at 25 °C and hydrogen permeability (P, cm^2^/s) at RH = 95% and t = 25 °C for the investigated membranes.

Membrane	$t_{Na^{+}}^{+},\;\%$	P·10^7^, cm^2^/s
M[IPA-H_2_O 80–20]	83.1 ± 1.3	1.92 ± 0.04
M[DMF]	85.0 ± 1.2	1.26 ± 0.04
M[NMP]	85.3 ± 1.4	1.27 ± 0.04

## Data Availability

Data might be available on request.
